# Morphology controlled synthesis of Fe^3+^-doped upconversion nanomaterials†

**DOI:** 10.1039/d3ra07908b

**Published:** 2024-02-07

**Authors:** Fuhua Huang, Cong Liu, Zhuoya Zhao, Li Wang, Jinglai Zhang, Hans Ågren, Jerker Widengren, Haichun Liu

**Affiliations:** a Hunan Provincial Key Laboratory of Environmental Catalysis & Waste Recycling, College of Materials and Chemical Engineering, Hunan Institute of Engineering Xiangtan 411104 P. R. China; b Henan Center for Outstanding Overseas Scientists, Henan University KaiFeng 475004 P. R. China; c College of Chemistry and Molecular Sciences, Henan University KaiFeng Henan 475004 P. R. China; d Henan Key Laboratory of Protection and Safety Energy Storage of Light Metal Materials, Henan University KaiFeng Henan 475004 P. R. China; e Department of Applied Physics, KTH Royal Institute of Technology S-10691 Stockholm Sweden haichun@kth.se

## Abstract

This work details the synthesis of paramagnetic upconversion nanoparticles doped with Fe^3+^ in various morphologies *via* the thermal decomposition method, followed by comprehensive characterization of their structures, optical properties and magnetism using diverse analytical techniques. Our findings demonstrate that by precisely modulating the ratio of oleic acid to octadecene in the solvent, one can successfully obtain hexagonal nanodiscs with a consistent and well-defined morphology. Further adjustments in the oleic acid to octadecene ratio, coupled with fine-tuning of the Na^+^/F^−^ ratio, led to the production of small-sized nanorods with uniform morphology. Significantly, all Fe^3+^-doped nanoparticles displayed pronounced paramagnetism, with magnetic susceptibility measurements at 1 T and room temperature of 0.15 emu g^−1^ and 0.14 emu g^−1^ for the nanodiscs and nanorods, respectively. To further enhance their magnetic properties, we replaced the Y-matrix with a Gd-matrix, and by fine-tuning the oleic acid/octadecene and Na^+^/F^−^ ratios, we achieved nanoparticles with uniform morphology. The magnetic susceptibility was 0.82 emu g^−1^ at 1 T and room temperature. Simultaneously, we could control the nanoparticle size by altering the synthesis temperature. These upconversion nanostructures, characterized by both paramagnetic properties and regular morphology, represent promising dual-mode nanoprobe candidates for optical biological imaging and magnetic resonance imaging.

## Abstract 2

This work details the synthesis of paramagnetic upconversion nanoparticles doped with Fe^3+^ in various morphologies *via* a thermal decomposition method, followed by comprehensive characterization using diverse analytical techniques.

## Graphical abstract

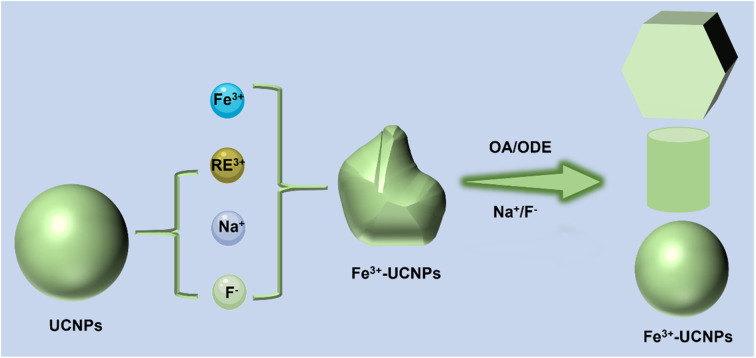

## 1 Introduction

Upconversion nanoparticles (UCNPs) possess the unique ability to convert low-energy into high-energy photons due to the interactions between the ample energy levels of the lanthanide dopants.^1^ UCNPs have found widespread applications in biomedical areas owing to their advantages, such as high photochemical stability, low toxicity and high light-conversion ability.^2–6^ Currently, UCNPs are primarily utilized as luminescent labeling materials for optical imaging.^7–9^ To expand their utility in multimodal applications, it is crucial to develop additional functionalities.

Magnetic nanomaterials represent a significant category within the realm of nanotechnology. Materials with magnetic properties often contain elements like iron, cobalt, and nickel. Magnetic nanomaterials play a vital role in nanomedicine.^10^ For instance, magnetic nanoparticles can be selectively concentrated in specific areas of an organism by using an external magnetic field, enhancing their spatial selectivity and efficacy.^11^ They can also respond to alternating magnetic fields, generating heat, thereby enabling magnetothermal therapy. When dispersed, superparamagnetic nanoparticles can also serve as contrast agents in magnetic resonance imaging (MRI).

Combining upconversion nanomaterials with magnetic materials presents an opportunity to develop magnetic properties of upconversion nanomaterials while preserving their excellent optical properties. This can lead to the creation of multifunctional upconversion magnetic nanomaterials with applications in biomedical imaging, magnetic medical diagnosis, and as parts in different treatment technologies. Currently, there are two main strategies for enhancing the magnetism of upconversion nanomaterials. The first strategy is to combine UCNPs with magnetic nanocrystals (*e.g.* Fe_3_O_4_) *via* the coating method or using organic crosslinkers as assistance.^11^ However, the nanocomposites produced using this strategy often suffer from difficulties in control, in producing large and uneven sizes, and poor product repeatability. The second strategy is to directly dope magnetically significant ions into the upconversion nanocrystals to confer magnetism to UCNPs. Recent studies have shown that nano-sized NaYF_4_: Yb, Er nanoparticles can be doped with transition metal ions, such as *e.g.* Fe^3+^, leading to the creation of ferromagnetic upconversion luminescent materials.^12^

Among magnetic ions, iron-containing materials are preferred due to their cost-effectiveness, availability, and non-toxic nature. Researchers have employed various synthesis methods to explore Fe^3+^-doped UCNPs, yielding meaningful results.^12–15^ However, the obtained nanomaterials often suffer from uneven size, irregular morphology, and limited controllability. For instance, Bai *et al.* synthesized CeO_2_: Fe, Yb, Er upconversion nanomaterials through a hydrothermal method, resulting in octahedral structures of approximately 120 nm edge-to-edge, composed of 3–5 nm small particles with non-uniform size and morphology.^16^ Nalupurackal *et al.* synthesized NaYF_4_: Yb, Er, Fe nanocrystals using a hydrothermal method, resulting in particles with a uniform morphology. However, their size (approximately 2 μm across) was relatively large.^12^ An *et al.* and Zhao *et al.* also employed a hydrothermal method to synthesize Fe^3+^-doped nanocrystals using NaYF_4_ and NaGdF_4_ as substrates, but both encountered issues with wide size distributions and poor morphology uniformity.^17,18^ Ramasamy *et al.* used a thermal decomposition method to synthesize NaGdF_4_: Yb, Er, Fe nanocrystals. This approach yielded uneven size distributions and spherical and elliptical morphologies.^15^ Similarly, Kumar *et al.* employed a thermal decomposition method to synthesize NaYF_4_: Yb, Tm, Er, Fe nanocrystals with a size of 26 nm, but faced challenges with uneven morphology.^13^ Additionally, Yamini *et al.* prepared NaGdF_4_: Yb, Er, Fe nanoparticles sized at 90 nm using a simple polyol method, resulting in irregular shapes, uneven sizes, and severe aggregation.^19^ In summary, the precise control of Fe^3+^ doping in UCNPs to achieve nanoparticles with controllable morphology, uniform size, and significant magnetism has remained an unsolved challenge.

In this work, we successfully engineered Fe^3+^-doped upconversion nanomaterials with precise control over their morphology and uniform particle size by meticulously manipulating the synthesis conditions by means of a thermal decomposition method. Comprehensive testing using various analytical techniques revealed that the incorporation of Fe^3+^ not only led to a marked enhancement in the luminescent efficiency of the upconversion material but also significantly bolstered its paramagnetic properties. This achievement establishes a robust framework for the controlled fabrication of Fe^3+^-doped NaYF_4_-/NaGdF_4_-based upconversion nanocrystals, setting the stage for further advancements in the development of UCNPs with even greater magnetic capabilities.

## 2 Experimental

### 2.1 Materials and reagents

Yttrium(iii) acetate hydrate (99.9%), ytterbium(iii) acetate hydrate (99.9%), erbium(iii) acetate hydrate (99.9%), and gadolinium(iii) acetate hydrate (99.9%) were purchased from Sigma-Aldrich. Sodium hydroxide (NaOH, >98%), ammonium fluoride (NH_4_F, >99.99%), 1-octadecene (ODE, 90%), and oleic acid (OA, 90%) were purchased from Aladdin®, China. Methanol (reagent grade), ethanol (reagent grade), and cyclohexane (reagent grade), and ferric nitrate (99.9%) were purchased from Sinopharm Chemical Reagent Co., China.

### 2.2 Synthesis of ∼150 nm NaYF_4_: 20% Yb, 2% Er, *x*% Fe (*x* = 0, 10, 20, 30) nanocrystals

All nanocrystals were synthesized using a previously reported protocol with modifications.^20^ NaYF_4_: 20% Yb, 2% Er, *x*% Fe (*x* = 0, 10, 20, 30) nanocrystals were synthesized in the following procedure. A total amount of 1 mmol RE(CH_3_COO)_3_ (RE = Y^3+^, Yb^3+^, Er^3+^) and Fe(NO_3_)_3_ was added into a mixture of oleic acid (9 mL) and 1-octadecene (12 mL) in a 250 mL flask at room temperature, and the mixture was heated to 160 °C and maintained for 30 min to form metal-oleate complexes. The resulting solution was cooled to room temperature, and then mixed with a methanol solution (10 mL) containing NH_4_F (4 mmol) and NaOH (2.5 mmol). After that, the temperature of the mixture was increased to 120 °C and maintained for 10 min for a complete methanol removal. The solution was then degassed for 15 min to remove residual methanol and oxygen. Subsequently, the temperature of the resulting solution was quickly increased to 300 °C and maintained for 90 min in an argon atmosphere. After the mixture cooled down to room temperature, the nanocrystals were precipitated with ethanol and collected by centrifugation at 10 000 rpm for 10 min. The products were re-washed with ethanol and centrifuged and finally re-dispersed in 10 mL of cyclohexane.

### 2.3 Synthesis of rod-like NaYF_4_: 20% Yb, 2% Er, *x*% Fe (*x* = 0, 10, 20, 30) nanocrystals

Rod-like NaYF_4_: 20% Yb, 2% Er, *x*% Fe (*x* = 0, 10, 20, 30) nanocrystals were synthesized following the below procedure. A total amount of 1 mmol RE(CH_3_COO)_3_ (RE = Y^3+^, Yb^3+^, Er^3+^) and Fe(NO_3_)_3_ was added into a mixture of oleic acid (20 mL) and 1-octadecene (15 mL) in a 250 mL flask at room temperature, and the mixture was heated to 160 °C and maintained for 30 min to form metal-oleate complexes. The resulting solution was cooled to room temperature, and then mixed with a methanol solution (10 mL) containing NH_4_F (4 mmol) and NaOH (6 mmol). After that, the temperature of the mixture was increased to 120 °C and maintained for 10 min for a complete methanol removal. Then the solution was degassed for 15 min to remove residual methanol and oxygen. Subsequently, the temperature of the resulting solution was quickly increased to 300 °C and maintained for 90 min in an argon atmosphere. After the mixture cooled down to room temperature, the nanocrystals were precipitated with ethanol and collected by centrifugation at 10 000 rpm for 10 min. The products were re-washed with ethanol and centrifuged and finally re-dispersed in 10 mL of cyclohexane.

### 2.4 Synthesis of NaGdF_4_: 20% Yb, 2% Er, *x*% Fe (*x* = 0, 10, 20, 30) nanocrystals

NaGdF_4_: 20% Yb, 2% Er, *x*% Fe (*x* = 0, 10, 20, 30) nanocrystals were synthesized by the following methods. A total amount of 1 mmol RE(CH_3_COO)_3_ (RE = Y^3+^, Yb^3+^, Er^3+^) and Fe(NO_3_)_3_ was added into a mixture of oleic acid (9 mL) and 1-octadecene (12 mL) in a 250 mL flask at room temperature, and the mixture was heated to 160 °C and maintained for 30 min to form metal-oleate complexes. The resulting solution was cooled to room temperature, and then mixed with a methanol solution (10 mL) containing NH_4_F (2.5 mmol) and NaOH (2.5 mmol). After that, the temperature of the mixture was increased to 120 °C and maintained for 10 min for a complete methanol removal. The solution was then degassed for 15 min to remove residual methanol and oxygen. Subsequently, the temperature of the resulting solution was quickly increased to 300 °C and maintained for 90 min in an argon atmosphere. After the mixture cooled down to room temperature, the nanocrystals were precipitated with ethanol and collected by centrifugation at 10 000 rpm for 10 min. The products were re-washed with ethanol and centrifuged and finally re-dispersed in 10 mL of cyclohexane.

### 2.5 Characterization

Scanning electron microscopy (SEM) images were obtained on a JEOL JSM-7610F Plus field emission scanning electron microscopy. Transmission electron microscopy (TEM) images were obtained on a JEOL JEM-2100 transmission electron microscope. The chemical states of the main components in the nanoparticles were determined by X-ray photoelectron spectroscopy (XPS), and the data were fitted using the “XPS Peak” software. Powder X-ray diffraction (XRD) patterns were collected on a Smart Lab diffractometer (Bruker, Germany) using Ni filtered Cu Kα radiation (*λ* = 0.154 nm). Magnetic susceptibility was tested using a magnetics measurement system produced by Quantum Design MPMS3. Upconversion luminescence spectra were recorded at room temperature on a HORIBA FluoroMax+ spectrofluorometer equipped with a fiber-coupled diode laser at 980 nm (Changchun New Industry).

## 3 Results and discussion

### 3.1 Synthesis of Fe^3+^-doped NaYF_4_-based paramagnetic upconversion nanodiscs with uniform shape and size

In the synthesis of paramagnetic nanodiscs, we utilized a thermal decomposition method with 1-octadecene (ODE) and oleic acid (OA) as the solvent and the surfactant, respectively.^13^ The ratio of OA to ODE plays a pivotal role in determining the size and shape of the resulting nanoparticles. For instance, Li and Chen *et al.* demonstrated a gradual transformation from spherical to rod-like nanoparticles by adjusting the OA-to-ODE ratio.^21,22^ Similarly, Shang *et al.* observed a transition from nanowires to hexagonal nanosheets with increasing OA dosage.^23^ Na *et al.* conducted a comprehensive analysis of the OA/ODE ratio's influence on the morphology of UCNPs, revealing that as the OA/ODE ratio increases, the nanoparticles exhibit a higher aspect ratio (AR), signifying increased anisotropy that impacts size and morphology.^24^

**Fig. 1:**
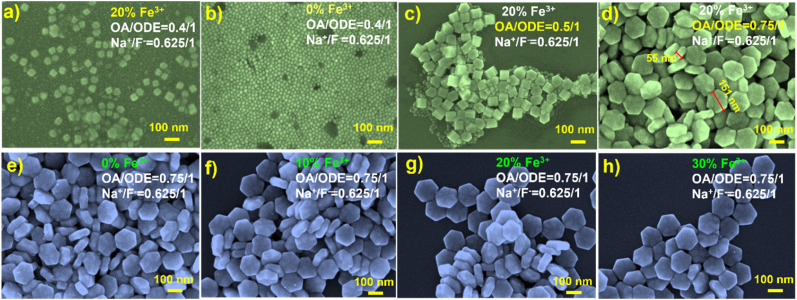
(a) SEM image of NaYF_4_: 20% Yb, 2% Er, 20% Fe nanoparticles, synthesized with OA/ODE = 0.4/1. (b) SEM image of NaYF_4_: 20% Yb, 2% Er nanoparticles, synthesized with OA/ODE = 0.4/1. (c) SEM image of NaYF_4_: 20% Yb, 2% Er, 20% Fe nanoparticles, synthesized with OA/ODE = 0.5/1. (d) SEM image of NaYF_4_: 20% Yb, 2% Er, 20% Fe nanoparticles, synthesized with OA/ODE = 0.75/1. (e) SEM image of NaYF_4_: 20% Yb, 2% Er, 0% Fe nanoparticles, synthesized with OA/ODE = 0.75/1. (f) SEM image of NaYF_4_: 20% Yb, 2% Er, 10% Fe nanoparticles, synthesized with OA/ODE = 0.75/1. (g) SEM image of NaYF_4_: 20% Yb, 2% Er, 20% Fe nanoparticles, synthesized with OA/ODE = 0.75/1. (h) SEM image of NaYF_4_: 20% Yb, 2% Er, 30% Fe nanoparticles, synthesized with OA/ODE = 0.75/1.

Among previous reports on the synthesis of 1 mmol NaYF_4_ nanocrystals, 0.4/1 (typically 6 mL OA and 15 mL ODE) has been commonly employed. In our initial attempts to synthesize Fe^3+^-doped upconversion nanoparticles (NaYF_4_: 20% Yb, 2% Er, 20% Fe), following this ratio, SEM characterization revealed irregular shapes, with most nanoparticles resembling cubes (Fig. 1a). In contrast, UCNPs synthesized under the same conditions without Fe^3+^ doping exhibited a uniform and consistently smaller size (Fig. 1b and S1†). This stark difference underscores the significant influence of Fe^3+^ doping on the growth of the UCNPs. While the OA/ODE ratio of 0.4/1 has been generally applicable for synthesizing UCNPs with various rare earth ion concentrations, this does not apply for Fe^3+^ doping. Consequently, we systematically adjusted the OA/ODE ratio to synthesize nanoparticles with the same Fe^3+^ doping concentration. SEM characterization revealed that as the OA/ODE ratio increased, the nanoparticles gradually transitioned from inhomogeneous to uniform (Fig. 1c and d). When the OA/ODE ratio reached 0.75/1, we successfully obtained hexagonal nanodiscs with regular shapes and uniform sizes (approximately 150 nm in diameter and 55 nm in thickness) (Fig. 1d). However, further increasing the OA/ODE ratio to 1/1 again resulted in non-uniform nanoparticles, including nanospheres of approximately 10 nm adhering to the hexagonal nanodiscs (Fig. S2a†). Ratios of 1.3/1 and 2/1 yielded even more inhomogeneous size distributions and reduced nanodisc thickness (Fig. S2b and c†). Thus, we determined the optimal OA/ODE ratio to 0.75/1, yielding more uniform and regular nanocrystals. The solvent proportion predominantly influences the UCNP morphology because the OA concentration affects the growth rate in different crystallographic directions, effectively promoting nanocrystal growth along the [101̄0] direction.^23,24^ Under the established optimal OA/ODE ratio, we synthesized a series of NaYF_4_: 20% Yb, 2% Er, *x*% Fe (*x* = 0, 10, 20, 30) nanoparticles, varying the Fe^3+^ concentration. Remarkably, altering the Fe^3+^ concentration did not significantly affect the morphology or size of the nanodiscs (Fig. 1e–h).

Furthermore, it is notable that the morphology of upconversion nanomaterials doped with Co^2+^ has also been influenced by the OA/ODE ratio, as evidenced in prior research. Heng *et al.* employed an OA/ODE ratio of 1/1, yielding non-uniformly shaped NaYF_4_-based UCNPs.^25^ Notably, Xia *et al.* utilized an OA/ODE ratio of 0.8/1, resulting in Co^2+^-doped nanoparticles with remarkably uniform and regular morphology, closely resembling the conditions applied in our study (0.75/1).^26^ This underscores the significant role of altering the OA/ODE ratio in tailoring the shape and size of transition-metal-ion-doped upconversion nanomaterials.

**Fig. 2:**
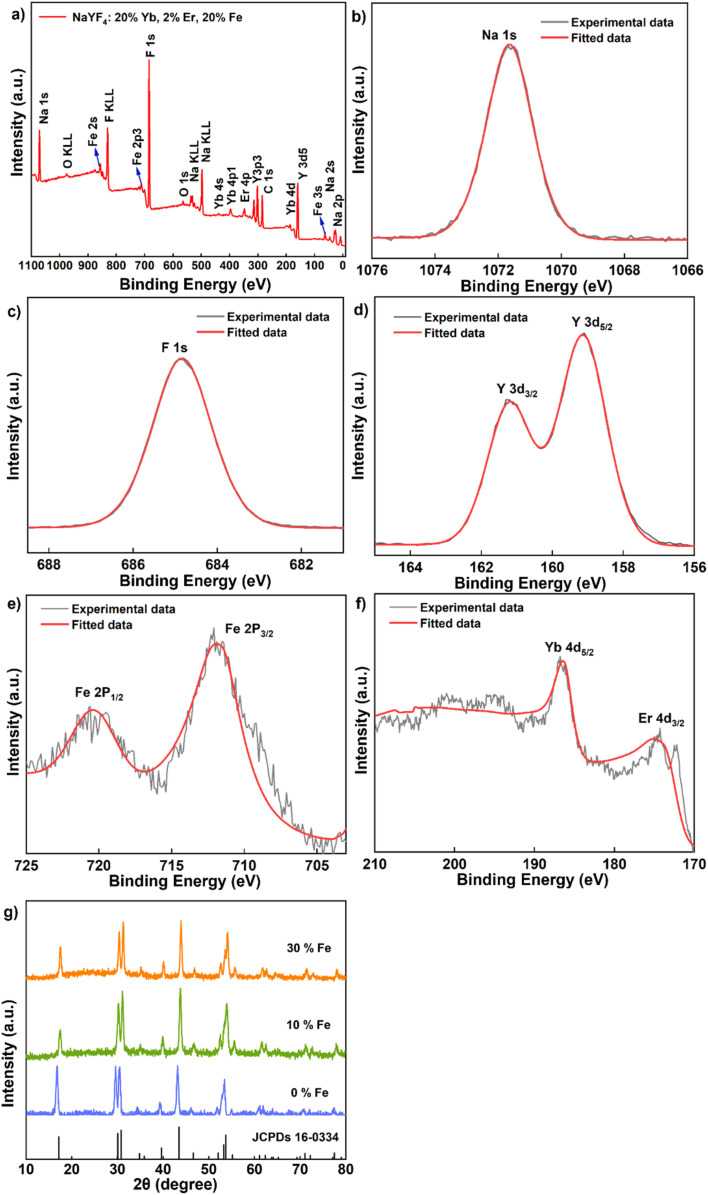
(a–f) XPS spectrum of the sample NaYF_4_: 20% Yb, 2% Er, 20% Fe. (g) XRD data of the samples NaYF_4_: 20% Yb, 2% Er, *x*% Fe (*x* = 0, 10, 30).

To confirm the successful Fe^3+^ doping, we conducted X-ray photoelectron spectroscopy (XPS) analyses on NaYF_4_: 20% Yb, 2% Er, 20% Fe (Fig. 2) nanoparticles. The XPS spectrum clearly revealed the presence of key elements, including Na, F, Y, Yb, Er and Fe (Fig. 2a). Specifically, the peak at 1071.5 eV corresponds to the binding energy of Na 1s (Fig. 2b), while the peak at 684.8 eV is attributed to the binding energy of F 1s (Fig. 2c). The peaks observed at 161 eV and 159 eV can be ascribed to the 3d_3/2_ and 3d_5/2_ peaks of Y, respectively (Fig. 2d). Furthermore, the peaks at 712.5 eV and 720.5 eV are indicative of the binding energy of Fe 2p_3/2_ and 2p_1/2_ (Fig. 2e), while the peaks at 174.5 eV and 186 eV represent the 4d levels of Er and Yb, respectively (Fig. 2f). To substantiate the atomic composition of NaYF_4_: 20% Yb, 2% Er, 20% Fe, additional elemental analysis (Y, Yb, Er, Fe) was conducted, as shown in Fig. S3.† These comprehensive results unequivocally confirm the successful incorporation of Fe^3+^ ions into the synthesized UCNPs, with a uniform distribution of all elements throughout the nanoparticles. To gain a more comprehensive understanding of the crystal structure, we conducted XRD measurements. In Fig. 2g, the test results for upconversion nanodiscs doped with 0%, 10% and 30% Fe^3+^ are depicted. These results reveal that the diffraction peaks of the samples closely matched the diffraction pattern of hexagonal NaYF_4_ lattice (JCPDS No. 16-0334), with no discernible impurity diffraction peaks. This suggests that the studied Fe^3+^ doping levels have no significant impact on the crystal phase of the nanoparticles.

**Fig. 3:**
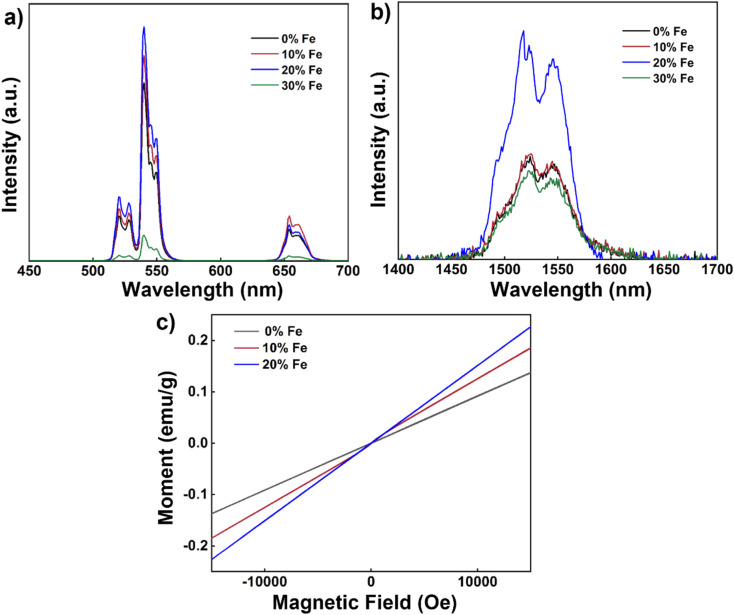
(a) Upconversion emission spectra of NaYF_4_: 20% Yb, 2% Er, *x*% Fe (*x* = 0, 10, 20, 30) nanoparticles (980 nm continuous-wave excitation, excitation power density: 2 W cm^−2^). (b) Down-conversion emission spectra of NaYF_4_: 20% Yb, 2% Er, *x*% Fe (*x* = 0, 10, 20, 30) nanoparticles (980 nm continuous-wave excitation, excitation power density: 2 W cm^−2^). (c) Magnetic susceptibility of NaYF_4_: 20% Yb, 2% Er, *x*% Fe (*x* = 0, 10, 20) nanoparticles at room temperature.

Next, we delved into the upconversion luminescence (UCL) properties of the nanoparticles. Fig. 3a displays the room temperature upconversion emission spectra of UCNPs doped with varying Fe^3+^ concentrations (ranging from 0 to 30 mol%) under identical excitation conditions (980 nm continuous wave excitation with an excitation power density of approximately 2 W cm^−2^). Notably, as the Fe^3+^ concentration increases from 0 mol% to 20 mol%, a marked enhancement in UCL intensity is observed. However, when the concentration reaches 30 mol%, the UCL intensity experiences a decline. Within this range of concentrations, 20 mol% Fe^3+^ ion doping emerges as the optimal concentration for augmenting UCL, with an intensity approximately 1.3 times that of undoped nanoparticles.

Similarly, under identical excitation conditions at 980 nm, the room temperature down-conversion emission spectra of the nanoparticles doped with different concentrations of Fe^3+^ (0–30 mol%) mirror the trend observed in the upconversion emission spectrum, with a noticeable increase in down-conversion luminescence (DCL) intensity as the Fe^3+^ ion concentration rises from 0 mol% to 20 mol% (Fig. 3b). Similarly, the intensity also diminishes as the Fe^3+^ ion concentration increases to 30 mol%. Again, 20 mol% Fe^3+^ ion doping proves to be the optimal concentration for enhancing DCL, with an intensity approximately 2.4 times that of undoped nanoparticles. To further explore the magnetic properties of the nanodiscs, we conducted magnetic susceptibility tests at room temperature, as depicted in Fig. 3c. The paramagnetism of the nanodiscs is progressively strengthened with increasing Fe^3+^ content. Specifically, the magnetic susceptibility of nanodiscs doped with 20% Fe^3+^ was found to be 0.15 emu g^−1^ at 1 T, marking a substantial enhancement compared to the corresponding undoped nanodiscs (0.09 emu g^−1^).

### 3.2 Controllable morphology and size tuning of Fe^3+^-doped NaYF_4_-based paramagnetic upconversion nanostructures

Numerous studies have demonstrated the critical influence of nanoparticle shape and size on their suitability for various biological applications.^27–30^ For instance, Tan *et al.* investigated the adhesion of nanoparticles, varying from spherical to short rod-shaped and long rod-shaped, with sizes of 100 nm and 200 nm, respectively, in blood vessels.^31^ Their findings revealed that long rod-shaped nanoparticles had a larger adhesion surface to the blood vessel wall compared to short rod-shaped ones. Furthermore, both rod-shaped nanoparticles exhibited higher adhesion than spherical nanoparticles, and smaller-sized nanoparticles displayed greater binding probabilities with cells compared to larger ones.^31^ Consequently, it is essential to exercise control over the shape and size of nanoparticles during the synthesis process.

In our previous work, by adjusting solvent ratios, we achieved uniform-sized hexagonal magnetic nanodiscs measuring approximately 150 nm side-to-side. However, while uniform in size, these nanodiscs present certain limitations for applications in the biological field. Firstly, their size exceeds the ideal range for biological use, where nanoparticles are generally preferred to be below 100 nm.^32^ Secondly, the hexagonal shape is less favored in the process of cellular endocytosis, compared to rod-shaped and spherical nanoparticles.^33,34^ Therefore, our objective is to regulate the shape and size of these hexagonal nanodiscs while preserving their optical and magnetic properties.

**Fig. 4:**
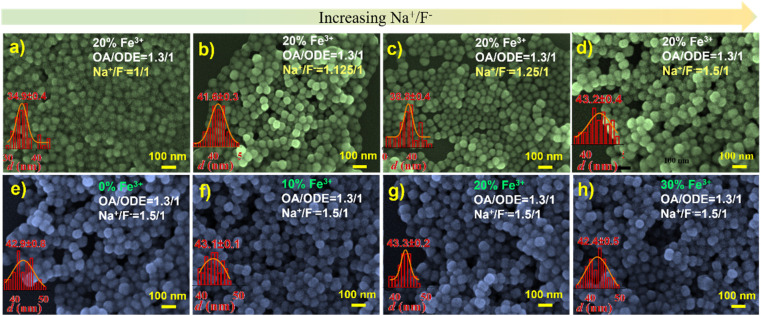
(a) SEM image of NaYF_4_: 20% Yb, 2% Er, 20% Fe nanoparticles, synthesized with Na^+^/F^−^ = 1/1. (b) SEM image of NaYF_4_: 20% Yb, 2% Er, 20% Fe nanoparticles, synthesized with Na^+^/F^−^ = 1.125/1. (c) SEM image of NaYF_4_: 20% Yb, 2% Er, 20% Fe nanoparticles, synthesized with Na^+^/F^−^ = 1.25/1. (d) SEM image of NaYF_4_: 20% Yb, 2% Er, 20% Fe nanoparticles, synthesized with Na^+^/F^−^ = 1.5/1. (e) SEM image of NaYF_4_: 20% Yb, 2% Er, 0% Fe nanoparticles, synthesized with Na^+^/F^−^ = 1.5/1. (f) SEM image of NaYF_4_: 20% Yb, 2% Er, 10% Fe nanoparticles, synthesized with Na^+^/F^−^ = 1.5/1. (g) SEM image of NaYF_4_: 20% Yb, 2% Er, 20% Fe nanoparticles, synthesized with Na^+^/F^−^ = 1.5/1. (h) SEM image of NaYF_4_: 20% Yb, 2% Er, 30% Fe nanoparticles, synthesized with Na^+^/F^−^ = 1.5/1.

We thus embarked on further exploration to fine-tune the controllable synthesis conditions of Fe^3+^-doped UCNPs. Thereby, we discovered that adjusting the OA/ODE to 1/1, while concurrently altering the Na^+^/F^−^ ratio during the synthesis process to 1/1 (typically 0.625/1), significantly reduces the size of these Fe^3+^-doped nanomaterials. This adjustment yielded hexagonal prisms measuring approximately 50 nm in diameter and about 43 nm in thickness, as illustrated in Fig. S4.† By further modifying the OA/ODE ratio to 1.3/1 adjusting the Na^+^/F^−^ ratio to 1.5/1 during synthesis, we observed a transformation in the shape of NaYF_4_: 20% Yb, 2% Er, 20% Fe nanoparticles from spherical (Fig. 4a) to progressively shorter rod-like structures (Fig. 4d). The TEM graph clearly depicts these short rod-shaped nanoparticles with a length of approximately 43 nm and a width of about 30 nm (Fig. S5†). These structural changes coincide with a Na^+^/F^−^ ratio of 1.5/1. Subsequent experiments using an OA/ODE ratio of 1.3/1 and a Na^+^/F^−^ ratio of 1.5/1 to manipulate the Fe^3+^ doping levels with the nanorods revealed no significant impact on their shape and size (Fig. 4e–h). In the UCNP synthesis, oleic acid molecules (OAH), as well as oleate (OA^−^) formed in the synthesis process, act as surfactants. OAH and OA^−^ can bind to different crystal facets with different binding affinities, related to their binding energies.^35^ Thus, varying the OA/ODE ratio can regulate the nanocrystals' growth in different directions. Adjusting the Na^+^/F^−^ ratio may change the chemical potential difference at different crystal facets, which also affects the microgrowth of the nanocrystals. Nevertheless, it must be acknowledged that the results obtained are empirical, lacking a discernible relationship between the OA/ODE and Na^+^/F^−^ ratios and the consistent morphology attained.

**Fig. 5:**
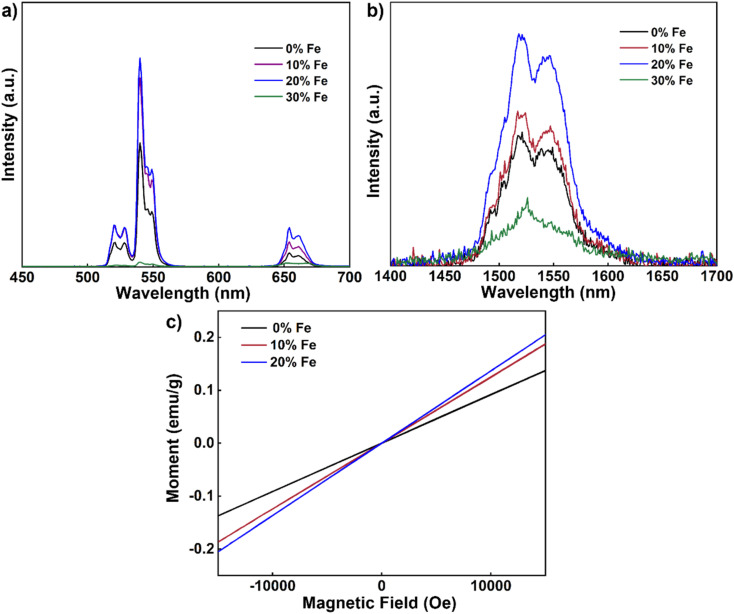
(a) Upconversion emission spectra of short rod-like NaYF_4_: 20% Yb, 2% Er, *x*% Fe (*x* = 0, 10, 20, 30) nanocrystals (980 nm continuous excitation, excitation power density: 2 W cm^−2^). (b) Down-conversion emission spectra of rod-like NaYF_4_: 20% Yb, 2% Er, *x*% Fe (*x* = 0, 10, 20, 30) nanocrystals (980 nm continuous excitation, excitation power density: 2 W cm^−2^). (c) Magnetic susceptibility of samples NaYF_4_: 20% Yb, 2% Er, *x*% Fe (*x* = 0, 10, 20) at room temperature.

To ascertain whether the nanorods hold promise for multimodal applications, we then conducted room temperature upconversion emission spectral tests on UCNPs co-doped with varying concentrations of Fe^3+^ ions (0–30 mol%) under excitation at 980 nm, as depicted in Fig. 5a. These emission spectra were recorded under consistent excitation conditions (continuous-wave excitation with an excitation power density of approximately 2 W cm^−2^). Notably, as the concentration of Fe^3+^ ions increased from 0 mol% to 20 mol%, a significant enhancement in UCL intensity was observed. However, when the concentration reached 30 mol%, the UCL intensity experienced a decrease. Consistent with our previous findings, a 20 mol% Fe^3+^ ion doping concentration emerged as optimal for augmenting UCL, with an intensity approximately 1.9 times that of undoped nanoparticles. Furthermore, we conducted tests on the down-conversion emission spectra at room temperature of UCNPs co-doped with varying concentrations of Fe^3+^ ions (0–30 mol%) under 980 nm excitation (Fig. 5b). Similarly to the upconversion spectrum, a noticeable increase in DCL intensity was observed with higher Fe^3+^ ion concentrations, from 0 mol% to 20 mol%. However, the DCL intensity diminished with further increased concentrations up to 30 mol%. Once again, 20 mol% Fe^3+^ ion doping emerged as the optimal concentration for enhancing DCL, with an intensity approximately 1.9 times that of undoped nanoparticles. It is known that lanthanide luminescence can be substantially affected by the change in the local crystal field induced by co-doping of other optically inert ions.^36,37^ Here the change in the UCL and DCL is likely due to the tailoring of the local environment around the lanthanide ions (Yb^3+^ and Er^3+^) induced by the substitution of Fe^3+^ ions with a small radius.

Next, we explored the magnetic susceptibility of the nanorods at room temperature, as illustrated in Fig. 5c. With increasing Fe^3+^ contents, the paramagnetic properties of the nanorods progressively became more pronounced. Notably, nanorods doped with 20% Fe exhibited a magnetic susceptibility of 0.14 emu g^−1^ at 1 T. These results underscore that smaller-sized nanorods exhibit enhanced magnetic properties after Fe^3+^ doping akin to their larger-sized nanodisc counterparts, opening up possibilities for multimodal applications of nanorods.

### 3.3 Synthesis of Fe^3+^-doped NaGdF_4_-based paramagnetic upconversion nanostructures

**Fig. 6:**
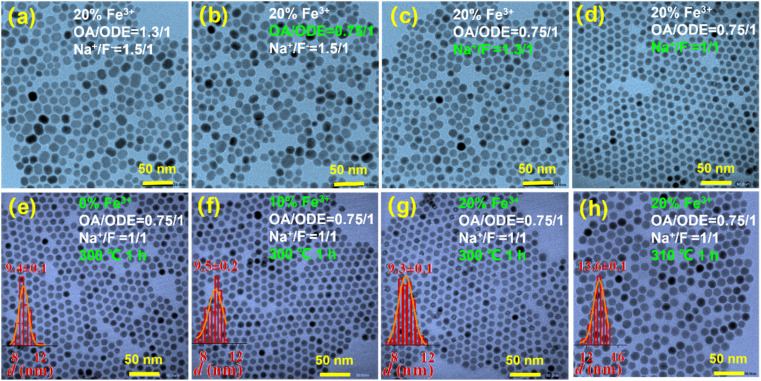
(a) TEM image of NaGdF_4_: 20% Yb, 2% Er, 20% Fe nanoparticles, synthesized with Na^+^/F^−^ = 1.5/1, OA/ODE = 1.3/1. (b) TEM image of NaGdF_4_: 20% Yb, 2% Er, 20% Fe nanoparticles, synthesized with Na^+^/F^−^ = 1.5/1, OA/ODE = 0.75/1. (c) TEM image of NaGdF_4_: 20% Yb, 2% Er, 20% Fe nanoparticles, synthesized with Na^+^/F^−^ = 1.3/1, OA/ODE = 0.75/1. (d) TEM image of NaGdF_4_: 20% Yb, 2% Er, 20% Fe nanoparticles, synthesized with Na^+^/F^−^ = 1/1, OA/ODE = 0.75/1. (e) TEM image of NaGdF_4_: 20% Yb, 2% Er, 0% Fe nanoparticles, synthesized with Na^+^/F^−^ = 1/1, OA/ODE = 0.75/1. (f) TEM image of NaGdF_4_: 20% Yb, 2% Er, 10% Fe nanoparticles, synthesized with Na^+^/F^−^ = 1/1, OA/ODE = 0.75/1. (g) TEM image of NaGdF_4_: 20% Yb, 2% Er, 20% Fe nanoparticles, synthesized with Na^+^/F^−^ = 1/1, OA/ODE = 0.75/1. (h) TEM image of NaGdF_4_: 20% Yb, 2% Er, 20% Fe nanoparticles, synthesized with Na^+^/F^−^ = 1/1, OA/ODE = 0.75/1, 310 °C, 1 h.

The magnetic properties of the Fe^3+^-doped NaYF_4_-based nanorods are still relatively weak, which makes their use in applications requiring strong magnetic properties challenging. We therefore opted for the substitution of Y^3+^ ions with Gd^3+^ ions, driven by the opportunity to leverage the substantial magnetic moment associated with Gd^3+^ ions due to a significant number of unpaired electron spins. Initially, we synthesized NaGdF_4_: 20% Yb, 2% Er, 20% Fe, following the nanorod reaction conditions depicted in Fig. 6a. However, the morphology of the resulting nanoparticles was inconsistent. Subsequently, we maintained a Na^+^/F^−^ ratio of 1.5/1 and reduced the OA/ODE ratio to 0.75/1. The TEM test results are displayed in Fig. 6b, where the morphology still exhibited irregularities with numerous rod-like particles. To address this, we retained the OA/ODE ratio at 0.75/1 and adjusted the Na^+^/F^−^ ratio to 1.3/1. TEM test results demonstrated a tendency toward uniformity, with a reduction in rod-like particles, as indicated in Fig. 6c. Maintaining the OA/ODE ratio at 0.75/1, we further adjusted the Na^+^/F^−^ ratio to 1/1. The TEM test results displayed uniformly spherical nanoparticles, as shown in Fig. 6d. Through this manipulation of the OA/ODE ratio and Na^+^/F^−^ ratio, we achieved homogeneous Gd matrix nanoparticles doped with Fe^3+^ ions. Additionally, increasing or decreasing the Fe^3+^ content ions in the nanoparticles at this stage did not significantly impact their shape and size, as evidenced in Fig. 6e–g.

At this point, the synthesized nanoparticles measured approximately 9.5 nm in diameter, owing to the influence of the Gd matrix. The nanoparticle size can be further adjusted by altering the synthesis temperature. As illustrated in Fig. 6h, elevating the reaction temperature from 300 °C to 310 °C for 1 h resulted in an increased nanoparticle size of 13.6 nm in diameter.

**Fig. 7:**
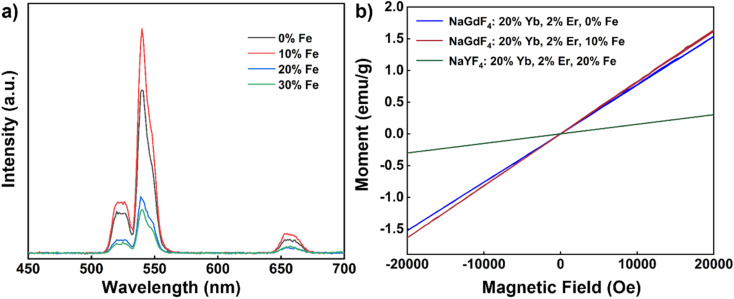
(a) Upconversion emission spectra of NaGdF_4_: 20% Yb, 2% Er, *x*% Fe (*x* = 0, 10, 20, 30) nanoparticles (980 nm continuous excitation, excitation power density: 2 W cm^−2^). (b) Magnetic susceptibility of NaGdF_4_: 20% Yb, 2% Er, 0% Fe, NaGdF_4_: 20% Yb, 2% Er, 10% Fe and NaYF_4_: 20% Yb, 2% Er, 20% Fe nanocrystals.

We also conducted room temperature upconversion emission spectrum tests on UCNPs co-doped with varying concentrations of Fe^3+^ ions (0–30 mol%), recorded under the same excitation conditions as above (980 nm continuous wave, excitation power density approximately 2 W cm^−2^), as shown in Fig. 7a. With increased Fe^3+^ ion concentrations from 0 mol% to 10 mol%, a noticeable enhancement in UCL intensity was observed. However, when the concentration increased to 20 mol%, the UCL intensity began to decline. Optimal UCL enhancement thus occurred at 10 mol% Fe^3+^ ion doping, with an intensity approximately 1.4 times greater than that of undoped nanoparticles.

Subsequently, we evaluated the magnetic susceptibility of the nanoparticles at room temperature, as shown in Fig. 7b. Compared with undoped Fe^3+^ nanoparticles, the magnetic susceptibility of NaGdF_4_: 20% Yb, 2% Er, 10% Fe is slightly improved after 10% Fe^3+^ ion doping. In comparison to NaYF_4_: 20% Yb, 2% Er, 20% Fe nanorods, the magnetic susceptibility of NaGdF_4_: 20% Yb, 2% Er, 10% Fe nanoparticles exhibited significant improvement. At 1 T and room temperature, the magnetic susceptibility of NaGdF_4_: 20% Yb, 2% Er, 10% Fe nanoparticles measured 0.82 emu g^−1^, which is 5.9 times higher than that of the aforementioned nanorods (Table 1). It is notable that the enhancement in the magnetic susceptibility by doping Fe^3+^ ions in the NaGdF_4_ matrix is not as significant as in the NaYF_4_ matrix. These findings demonstrate that, through adjustments in synthesis conditions, it is possible to obtain Fe^3+^ ion-doped nanoparticles with regular morphology and uniform size with the NaGdF_4_ matrix. This enhancement in paramagnetic properties opens up exciting possibilities for multimodal applications of these nanoparticles.

**Table 1:** Magnetic comparison of the three nanoparticles at 1 T and room temperature

Sample	Size (nm)	Magnetization (emu g^−1^)
NaYF_4_: 20% Yb, 2% Er, 20% Fe	Diameter: 150	0.15
	Thickness: 55	
NaYF_4_: 20% Yb, 2% Er, 20% Fe	Length: 43	0.14
	Width: 30	
NaGdF_4_: 20% Yb, 2% Er, 10% Fe	Diameter: 9.5	0.82

## 4 Conclusion

In the present work we synthesized Fe^3+^-doped upconversion nanodiscs with regular morphology and uniform size distribution by finely tuning the OA/ODE ratio in the solvent using a thermal decomposition method. Through optical and magnetic performance tests, we observed that these nanodiscs exhibit robust paramagnetic properties and a pronounced enhancement in UCL. To broaden the application potential of these multifunctional nanoparticles, we also produced nanorods with consistent size and regular shape by making precise adjustments to the OA/ODE and Na^+^/F^−^ ratios during the synthesis process. Our investigations revealed that these Fe^3+^-doped nanorods also exhibit favorable UCL properties and paramagnetism.

To further enhance their magnetic properties, we replaced the Y-based matrix with a Gd-based matrix, and by fine-tuning the OA/ODE and Na^+^/F^−^ ratios we achieved also then nanoparticles with uniform morphology. Simultaneously, we could control the nanoparticle size by altering the synthesis temperature. Our work yields valuable insight into the controlled synthesis of size- and morphology-tailored Fe^3+^-doped UCNPs. However, it is essential to note that the addition of Fe^3+^ during UCNP synthesis significantly impacts the microgrowth process of the nanocrystals, and the optimal OA/ODE and Na^+^/F^−^ ratios for rare earth-doped UCNP synthesis may so not be ideal for synthesis of all Fe^3+^-doped materials. This presents a challenge for achieving precise controllable synthesis. It is also notable that, compared to iron oxide nanomaterials, the magnetic susceptibility of these materials remains relatively weak and requires further enhancement to meet the demands of most applications reliant on magnetic properties. Nevertheless, our research demonstrates the feasibility of finding the delicate balance in OA/ODE and Na^+^/F^−^ ratios to enable size-tunable, uniform and controlled synthesis of Fe^3+^-doped UCNPs and sets the groundwork for advancing magnetic upconversion nanomaterials.

## Conflicts of interest

There are no conflicts of interest to declare.

## Supplementary Material

RA-014-D3RA07908B-s001
