# Supplementary material for: Morphology controlled synthesis of Fe3+-doped upconversion nanomaterials
Source: RSC Adv. 2024 Feb 7;14(8):4990–5000. doi: 10.1039/d3ra07908b (PMC10848240; doi:10.1039/d3ra07908b)
Supplement: RA-014-D3RA07908B-s001 [file RA-014-D3RA07908B-s001.pdf]

Electronic Supplementary Material (ESI) for RSC Advances.
This journal is © The Royal Society of Chemistry 2024

Supplementary Information

Morphology controlled synthesis of Fe^{3+}-doped upconversion nanomaterials

Fuhua Huang[1,2,3,4], Cong Liu[2,3,4], Zhuoya Zhao[2,3,4], Li Wang[2,3,4], Jinglai Zhang[2,3,4], Hans Ågren[2,3,4], Jerker Widengren[5], Haichun Liu*[5]

[1] *Hunan Provincial Key Laboratory of Environmental Catalysis & Waste Recycling, College of Materials and Chemical Engineering, Hunan Institute of Engineering, Xiangtan 411104, P. R. China*

[2] *Henan Center for Outstanding Overseas Scientists, Henan University, KaiFeng 475004, P. R. China*

[3] *College of Chemistry and Molecular Sciences, Henan University, KaiFeng, Henan 475004, P. R. China*

[4] *Henan Key Laboratory of Protection and Safety Energy Storage of Light Metal Materials, Henan University, KaiFeng, Henan 475004, P. R. China*

[5] *Department of Applied Physics, KTH Royal Institute of Technology, S-10691, Stockholm, Sweden*

Supplementary Figures

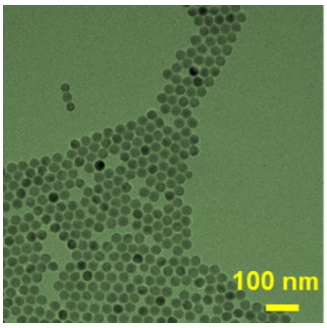

Fig. S1 TEM image of $NaYF_4$: 20%Yb, 2%Er nanoparticles, synthesized with OA/ODE=0.4/1.

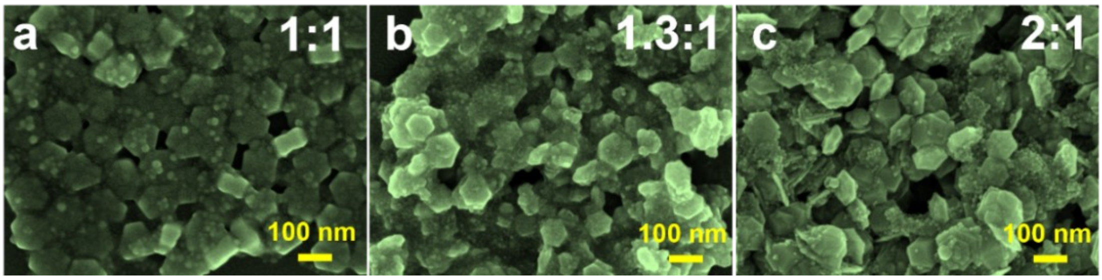

Fig. S2 SEM images of $NaYF_4$: 20% Yb, 2% Er, 20% Fe nanoparticles, synthesized with (a) OA/ODE=1/1, (b) OA/ODE=1.3/1, and (c) OA/ODE=2/1.

---

*Corresponding author.
E-mail addresses: haichun@kth.se (H. Liu)

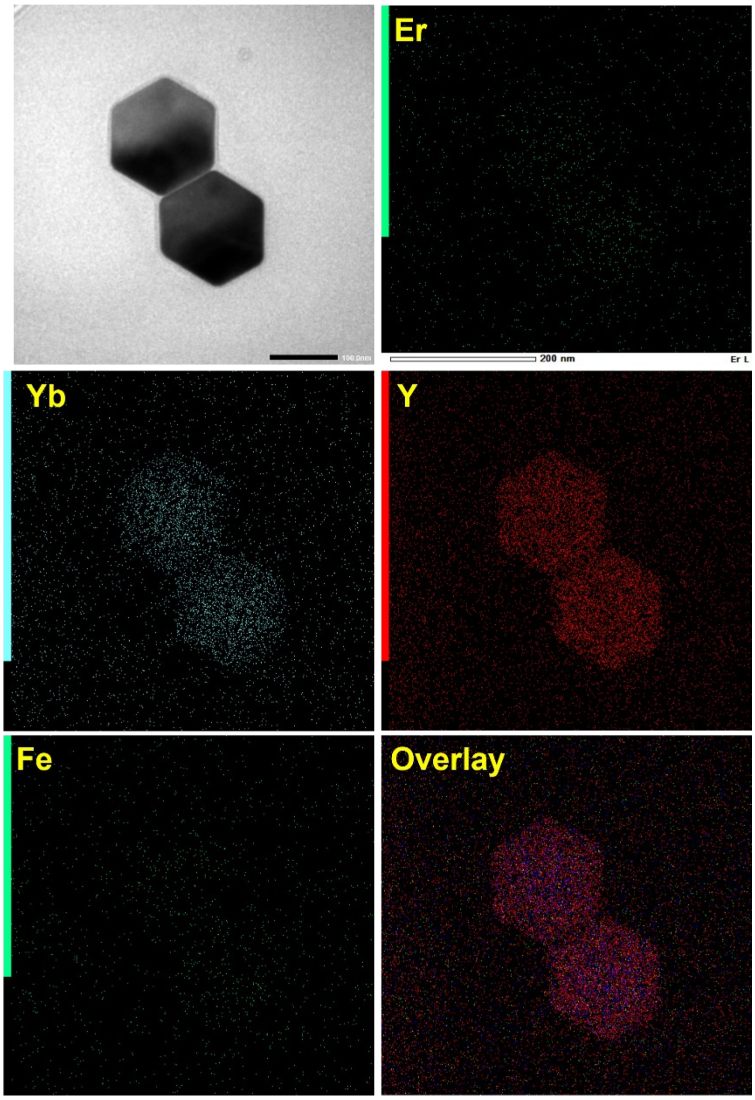

Fig. S3 Elemental analysis chart of $NaYF_4$: 20% Yb, 2% Er, 20% Fe nanocrystals.

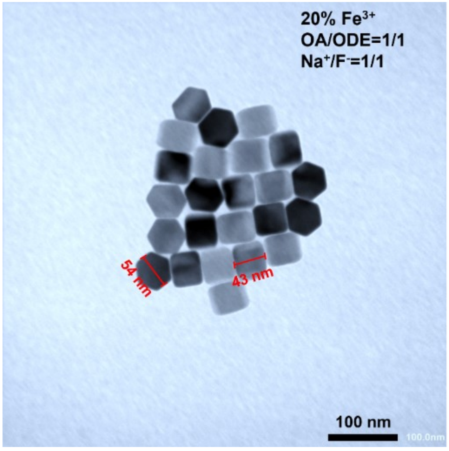

Fig. S4 TEM image of $NaYF_4$: 20% Yb, 2% Er, 20% Fe nanoparticles, synthesized with OA/ODE=1/1 and Na^+/F^-=0.625/1.

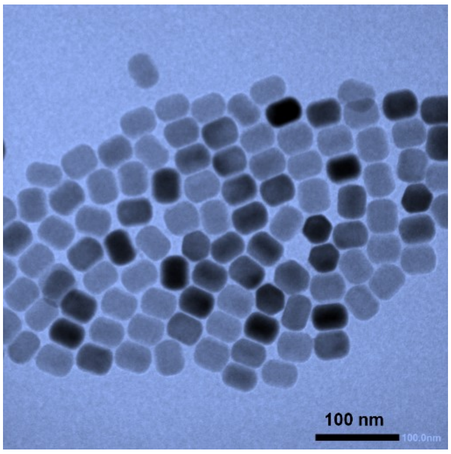

Fig. S5 TEM image of $NaYF_4$: 20% Yb, 2% Er, 20% Fe nanoparticles, synthesized with OA/ODE=1.3/1 and Na^+/F^-=1.5/1.
